# Genome-Wide Identification and Expression Analysis of *VviYABs* Family Reveal Its Potential Functions in the Developmental Switch and Stresses Response During Grapevine Development

**DOI:** 10.3389/fgene.2021.762221

**Published:** 2022-02-03

**Authors:** Songtao Jiu, Yanping Zhang, Peng Han, Yubo Han, Yan Xu, Gengsen Liu, Xiangpeng Leng

**Affiliations:** ^1^ Department of Plant Science, School of Agriculture and Biology, Shanghai Jiao Tong University, Shanghai, China; ^2^ Suzhou Polytechnic Institute of Agriculture, Suzhou, China; ^3^ Jiangbei Grape Research Institute of Shandong Province, Shandong, China; ^4^ Institute of Grape Science and Engineering, College of Horticulture, Qingdao Agricultural University, Qingdao, China

**Keywords:** grapevine, genome-wide analysis, *VviYABs*, berry development, abiotic and biotic stresses

## Abstract

Plant-specific YABBY (YAB) transcription factors play multiple roles in plant growth and development process. However, no comprehensive study has been performed in grapevines, especially to determine their roles in berry development and abiotic stress response. A total of seven *VviYABs* allocated to six chromosomal positions in grapevines were identified and classified into five subfamilies based on phylogenetic and structural analysis. Promoter element analysis and tissue-specific transcriptional response of *VviYABs* suggested that *VviYABs* might play vital roles in plant growth and development. *VviYAB1*, *2*, *3*, and *5* showed significantly higher expression levels in vegetative/green organs than in mature/woody tissues, implying that *VviYABs* might be involved in the regulatory switch from immature to mature developmental phases. The expression of *VviYAB1*, 2, 3, and *VviFAS* were gradually downregulated during berry developmental and ripening, which can be considered as putative molecular biomarkers between vegetative/green and mature/woody samples, and were used to identify key developmental and metabolic processes in grapevines. Furthermore, *VviYAB1* expression was not markedly increased by gibberellic acid (GA_3_) treatment alone, but displayed significant upregulation when GA_3_ in combination with N-(2-chloro-4-pyridyl)-N′-phenylurea (CPPU) were applied, suggesting an involvement of *VviYAB1* in fruit expansion by mediating cytokinin signaling pathway. Additionally, microarray and RNA-seq data suggested that *VviYABs* showed transcriptional regulation in response to various abiotic and biotic stresses, including salt, drought, *Bois Noir*, *Erysiphe necator*, and GLRaV-3 infection. Overall, our results provide a better understanding of the classification and functions of *VviYABs* during berry development and in response to abiotic and biotic stresses in grapevines.

## Introduction

YABBY (YAB) is a small transcription factor gene family, which is specific seed plants (Bowman and Smyth, 1999; Shamimuzzaman and Vodkin, 2013). The YAB genes consist of two conserved domains: an N-terminal zinc-finger motif and a C-terminal YABBY domain (helix–loop–helix motif) (Golz and Hudson, 1999; Siegfried et al., 1999). In *Arabidopsis*, *YAB* genes specify abaxial cell fate in lateral organs (Villanueva et al., 1999; Bowman, 2000). However, several reports have shown that *YABs* in monocots have evolved diverse functions in other developmental processes (Liu et al., 2007; Yang et al., 2016).

The *Arabidopsis* genome comprises six *YAB* genes, which are divided into five subfamilies: CRABS CLAW (CRC), FILAMENTOUS FLOWER (FIL)/YAB3, INNER NO OUTER (INO), YAB2, and YAB5 (Bowman, 2000; Eckardt., 2010; Sarojam et al., 2010). Among them, *FIL*, *YAB2*, *YAB3*, and *YAB5* function redundantly to control the development of lateral organs (Siegfried et al., 1999; Yamada et al., 2011; Yang et al., 2018). In *Arabidopsis*, *FIL* is responsible for the flower formation and development (Chen et al., 1999; Sawa et al., 1999a, Sawa et al., 1999b). Similarly, the *TOB1*-related *YAB* genes in rice have conserved functions in flower development(Tanaka et al., 2017). The *CRC* gene in *Arabidopsis* promotes carpel polarity and nectary development (Alvarez and Smyth, 1999), and its orthologs in rice and *Pisum sativum* also regulate the carpel morphogenesis (Yamaguchi et al., 2004; Fourquin et al., 2014). *INO* plays a crucial role in the regulation of the outer integument growth (Villanueva et al., 1999). In rice, both *OsYAB1* and *OsYAB4* participate in controlling the gibberellin pathway, and overexpression of the rice *OsYAB1* and *OsYAB4* leads to a semi-dwarf phenotype and increases in the number of stamens (Dai et al., 2007; Yang et al., 2016).

*YAB* genes participate in fruit morphogenesis during fruit development. For example, downregulation of the *FASCIATED* (*FAS*) gene, encoding a YAB transcription factor, results in an increase of locule number in modern tomato varieties (Cong et al., 2008). In grapevines, *VviYAB5* displays different expression patterns between tricarpellate and bicarpellate ovaries, indicating that the expression of *VviYAB5* is associated with the formation of tricarpellate fruit (Xing et al., 2016). Furthermore, growing evidences show that *YAB* genes play important roles in plant abiotic stress responses (Inal et al., 2017; Zhao et al., 2017). For example, the soybean *GmYAB10* negatively regulates the drought and salt tolerance (Zhao et al., 2017).

The grapevine is among the most globally cultivated and economically important fruit crops worldwide (Leng et al., 2017; Jiu et al., 2021; Jiu et al., 2022; Xu et al., 2021). A preliminary study in *Vitis pseudoreticulata* showed that *VpYAB1* was involved in leaf development, and ectopic expression of *VpYAB1* led to the loss of dorso-ventral polarity in the leaf blade (Xiang et al., 2013). However, comprehensive investigations are needed to evaluate the function of YAB gene family in *Vitis vinifera*. In the present study, we observed the genomic organization and transcript patterns of the *YAB* members in grapevines and focused on the roles of *VviYAB* genes in fruit development and stress response. Our results will facilitate further investigation into the functions of the *VviYAB* and may have potential improvement for molecular breeding programs in grapevines.

## Materials and Methods

### Identification and Analysis of *VviYABs* Family in Grapevines

The hidden Markov model (HMM) profile of the YABBY (PF04690) was downloaded from the Pfam database (http://pfam.xfam.org/) and used to survey the grapevine genome database (CRIBI; http://genomes.cribi.unipd.it/grape/) using HMMER3.1 as in previous reports (Leng et al., 2019). All protein sequences of VviYABs were identified based on the YABBY domain using Simple Modular Architecture Research Tool (SMART) database (http://smart.embl-heidelberg.de/) and National Center for Biotechnology Information-Conserved Domain Database (NCBI-CDD) search (https://www.ncbi.nlm.nih.gov/cdd www.ncbi.nlm.nih. gov/cdd). The physical and chemical parameters as well as subcellular location of VviYABs were determined using ProtParam (http://web.expasy.org/protparam/) and WoLF PSORT (https://www. genscript.com/wolf-psort.html), respectively. These members of the *VviYABs* family were named according to the rules described by Grimplet et al. (2014).

### Sequence Alignments and Phylogenetic Analysis

Sequences of *Arabidopsis* and tomato YAB proteins were retrieved from The Arabidopsis Information Resource (TAIR; https://www.arabidopsis.org/) and the Solanaceae Genomics Network (https://solgenomics.net/), respectively. Multiple sequence alignments of these YAB proteins were performed using ClustalX. A phylogenetic tree based on the full-length amino acid (aa) sequences was constructed by using MEGA 5.0 with the neighbor-joining (NJ) method. Motif analyses of VviYABs were performed by using MEME tool (http://meme.sdsc.edu/meme/website/intro.html) as in previous reports (Wei et al., 2020).

### Chromosomal Locations, Gene Structure, and Gene Duplication

Chromosomal positions of *VviYAB* genes were obtained from the Grape Genome CRIBI website (http://genomes.cribi.unipd.it/). Exon–intron structures were determined using the Gene Structure Display Server (GSDS; http://gsds.cbi.pku.edu.cn) by comparing the cDNA sequences with their corresponding genomic sequences. Tandem duplicated genes were identified as adjacent paralogous on an individual grape chromosome, with no more than one intervening gene (Wei et al., 2020). The syntenic blocks within the grape genome, as well as among grapevines, *Arabidopsis*, and tomato, were detected using MCScanX software according to our previous research (Leng et al., 2019; Jiu et al., 2020).

### Promoter Analysis

The nucleotide sequences of *VviYAB* genes were obtained from the grapevine genome database (CRIBI; http://genomes.cribi.unipd.it/grape/) in this study. The upstream 1,500-bp region from the translation initiation site for all *VviYABs* was regarded as the putative promoter sequence (Badawi et al., 2019; Feng et al., 2021). The *cis*-acting elements of *VviYAB* genes were predicted using PlantCARE tool (http://bioinformatics.psb.ugent.be/webtools/plantcare/html/; Postel et al., 2002).

### Expression Patterns of *VviYABs* in Various Organs and Different Berry Developmental Stages

The microarray expression atlas of *VviYAB* genes in various organs and developmental stages were acquired from the Gene Expression Omnibus (GEO) datasets (GSE36128; Fasoli et al., 2012). The average expression value of each gene in all tested organs were analyzed and graphically drawn with MeV software (Saeed et al., 2006).

The transcriptional expression patterns of *VviYAB* genes at 13 different fruit developmental stages were collected from GEO datasets (GSE98923; Fasoli et al., 2018). Furthermore, other transcriptional expression analyses based on RNA-seq data were downloaded from GEO datasets (GSE62744 and GSE62745), which contained 10 grapevine varieties at four berry development stages (Massonnet et al., 2017).

### Plant Growth Condition, Hormone, and Stress Treatment

Four-year-old “Fujiminori” grapevines (*V. vinifera* × *V. labrusca*) were used as the experimental material, which were maintained in the Qingdao Agricultural University fruit farm, Qingdao, China. Eight grapevine samples, including young leaf, senescent leaf, green stem, woody stem, bud burst (green tip), winter bud, berry post-fruit set, and berry ripening were collected and used for tissue-specific expression. Furthermore, grapevine berries were collected at four developmental stages, including the pea-sized berry stage at 20 days after flowering (DAF, pea_sized), the berries beginning just prior to veraison (pre_veraison), the berry-softening at the end of veraison (end_veraison), and the fully ripe berry stage at harvest (ripe). Three biological replicates were used for different developmental stages of grapevine organs.

For abscisic acid (ABA) and ethylene treatments, 60 DAF of grape berries were immersed in 100 mg/L ABA and 500 mg/L ethephon (ETH, an ethylene-releasing reagent), and the control berries were immersed in deionized water. Berries were sampled at 0 (green), 10 (pre_veraison), 20 (end_veraison), and 30 days (ripening) after treatment. Three biological replicates were used for different harvest dates of ABA and ethylene treatments. All samples were immediately frozen and stored at −80°C until use. For gibberilic acid (GA_3_) and forchlorfenuron (CPPU) treatment, grapevine RNA-seq datasets were retrieved from published supplemental data sets (Xu et al., 2019), which contained three treatments, i.e., water (CK), 25 mg/L GA_3_ + 0 mg/L CPPU, and 25 mg/L GA_3_ + 10 mg/L CPPU (GA_3_ + CPPU), at full-bloom stage.

Expression analyses in response to abiotic and biotic stresses were downloaded from the NCBI GEO datasets based on microarray data (series matrix accession numbers GSE31594, GSE31677, GSE6404, GSE12842, and GSE31660). To further validate the expression profiles of *VviYABs* in response to different abiotic stress treatments, grapevine RNA-seq data in response to waterlogging and drought stress were retrieved from NCBI database (SRA accession no. SRP070475 and SRP074162, respectively) (Haider et al., 2017; Zhu et al., 2018). RNA-seq data for expression profiles in response to salt were retrieved from published supplemental datasets (Guan et al., 2018).

### qRT-PCR Analysis

Total RNA samples were isolated using the modified cetyltrimethyl ammonium bromide (CTAB) method (Leng et al., 2015a; Leng et al., 2015b; Jiu et al., 2019). Subsequently, 1.0 μg of total RNA was used to synthesize the first-strand cDNA using the Prime Script RT reagent Kit (TaKaRa Biotechnology, Dalian, China), and the resulting cDNA was diluted 10-fold before performing qRT-PCR assay. qRT-PCR reactions (20 μl) contained 10 μl SYBR Green Supermix (Bio-Rad), 0.4 μl 10 μM forward primer, 0.4 μl 10 μM reverse primer, and 2 μl cDNA template. The mixture was placed in an Applied Biosystems (ABI) 7500 FAST Real-Time PCR System, and the amplification was conducted using the program described by (Jiu et al. 2016, Jiu et al., 2021). Each pair of qRT-PCR primers was validated by cloning and sequencing of the product using this pair of primers. Each reaction was performed in triplicate. *Actin* (AB073011) was used as an internal standard to normalize the expression levels. Relative expression levels were estimated using the 2^–ΔΔCT^ method (Livak and Schmittgen, 2001). The gene-specific primers for the *VviYAB* genes are listed in Supplementary Table S1.

### Subcellular Localization

The full-length cDNA of the *VviCRC*, *VviFAS*, and *VviYAB3* were amplified using the primers (Supplementary Table S1) and cloned into the binary vector pHB including two cauliflower mosaic virus (CaMV) 35S promoter, a translation enhancer, and a green fluorescence protein (GFP) fluorescent protein tag, respectively, to generate three fusion constructs (35S-VviCRC-GFP, 35S-VviFAS-GFP, and 35S-VviYAB3-GFP). After the three identified sequences, the control vector (pHB) and three fusion constructs were transformed into *Agrobacterium tumefaciens* GV3101 strains and subsequently agroinfiltrated into the leaves of 3- to 5-week-old *Nicotiana benthamiana* plants. Localization of fluorescent proteins were observed 3–5 days after infiltration using a confocal laser scanning microscope (Zeiss LSM 780, Germany) according to the manufacturer's instructions.

### Statistical Analysis

The experiment was performed using a completely randomized design with three biological replicates. The data were statistically analyzed using the SAS software package (Version 9.2, SAS Institute Inc., Cary, NC, United States). For the qRT-PCR analysis, data are shown as means ± standard deviation (SD) of three biological replicates.

## Results

### Identification and Analysis of Full-Length *VviYABs* in Grapevines

To identify the *YABs* from the grapevine genome, local BLAST and HMM tools were used as described in previous studies (Leng et al., 2019; Wei et al., 2020). According to the presence of conserved YABBY domain, seven *VviYABs* were identified in the grapevine and named based on their homology to the *Arabidopsis* and tomato YAB members (Table 1). All seven *VviYABs* were unevenly distributed to 6 out of 19 grapevine chromosomal positions (Supplementary Figure S1). Detailed parameters of VviYAB proteins are listed in Table 1. Grapevine VviYAB proteins varied from 168 (VviCRC) to 211 (VviYAB3) aa in length. The grand average of hydropathicity (GRAVY) is less than 0 for all VviYAB proteins, implying that YAB proteins possess a hydrophilic nature (Table 1).

**TABLE 1:** Structural and biochemical information of identified *VviYABs* in grapevine.

Gene name	Accession no	Len (aa)	Chrom	Chr start	Chr end	MW (kDa)	*pI*	Aliphatic index	GRAVY	Loc
*VviYAB1*	*VIT_15s0048g00550.t01*	210	chr15	14675136	14677631	23199.61	8.57	77.05	−0.288	Nucl (7), Cyto (5), Plas (1)
*VviYAB2*	*VIT_08s0032g01110.t01*	184	chr8	5502674	5509282	20419.18	8.68	70.05	−0.395	Chlo (6), Nucl (5), Cyto (2)
*VviYAB3*	*VIT_02s0154g00070.t01*	211	chr2	4862199	4864424	23336.57	7.71	79.95	−0.293	Nucl (12), Cyto (1)
*VviYAB5*	*VIT_11s0016g05590.t01*	185	chr11	5014314	5017431	20818.72	8.43	73.35	−0.384	Extr (4), Nucl (2), Cyto (2), ER (2)
*VviFAS*	*VIT_06s0009g00880.t01*	183	chr6	11951344	11957729	20383.17	8.38	66.67	−0.461	Extr (4), ER (3), Cyto (2), Chlo (1)
*VviINO*	*VIT_01s0127g00330.t01*	176	chr1	7691531	7692677	19559.35	7.03	74.26	−0.386	Chlo (3), Extr (3), ER (3), Nucl: (2)
*VviCRC*	*VIT_01s0011g00140.t01*	168	chr1	237670	239297	18533.09	8.81	64.40	−0.518	Nucl (13)

Len, length; aa, amino acid; Chrom, chromosome; GRAVY, grand average of hydropathicity; Loc, subcellular location; MW, molecular weight; pI, theoretical isoelectric point. The subcellular localizations of grapevine VviYABs were predicted using WoLF PSORT (https://www.genscript.com/wolf-psort.html). Chlo, chloroplast; Cyto, cytosol; ER, endoplasmic reticulum; Extr, extracellular; Nucl, nucleus; Plas, plasma membrane. The numbers in parentheses indicate prior possible localization sites of the VviYAB protein.

### Phylogenetic, Structural, and Synteny Analysis of *VviYAB* Genes

**FIGURE 1:**
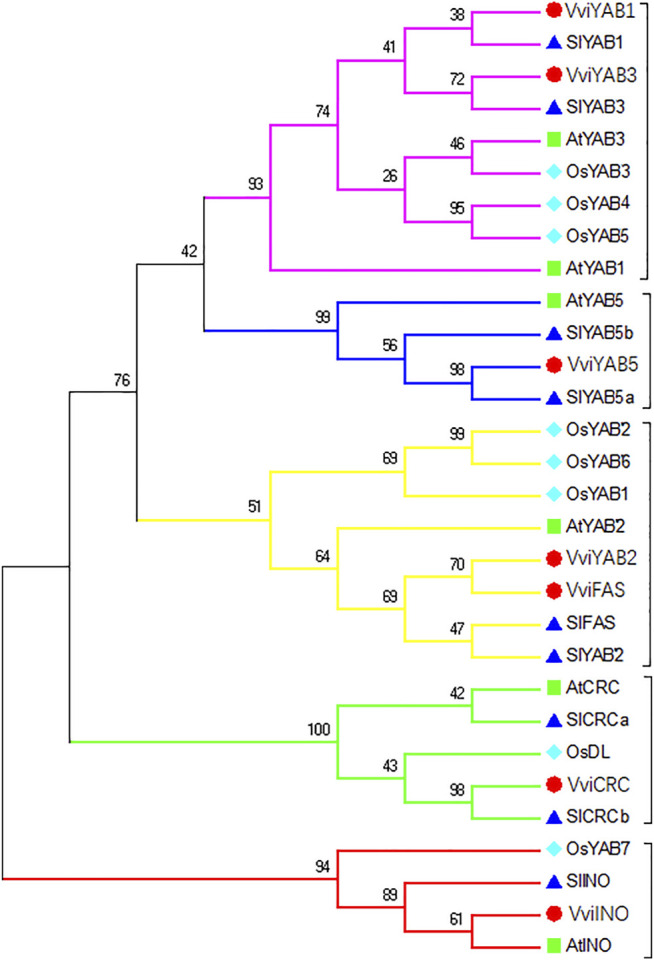
Phylogenetic relationships of YAB genes among four plant species. YABs were divided into five subfamilies (YAB1/YAB3, YAB2, YAB5, CRC, and INO) The neighbor-joining method was used, and the bootstrap values were set at 1,000.

To understand the evolutionary relationship between YAB proteins, a phylogenetic tree consisting of 30 YAB proteins from four different species was constructed using MEGA (7.0) (Figure 1). As shown in Figure 1, YAB members were further divided into five subgroups (CRC, INO, YAB1/YAB3, YAB2, and YAB5), which is consistent with previous reports in *Arabidopsis* and tomato (Bowman, 2000; Huang et al., 2013). *VviYABs* showed a closer relationship to dicotyledons (*Arabidopsis* and tomato) than monocotyledons (rice). Interestingly, no proteins from rice, the monocotyledon species, was found in the YAB5 subgroup, suggesting that *YABs* have diversified in the evolution of different species. In addition, a maximum of YAB proteins was observed in the YAB1/YAB3 subgroup, indicating that the YAB1/YAB3 subgroup was primarily attributed to the expansion of the YAB gene family.

**FIGURE 2:**
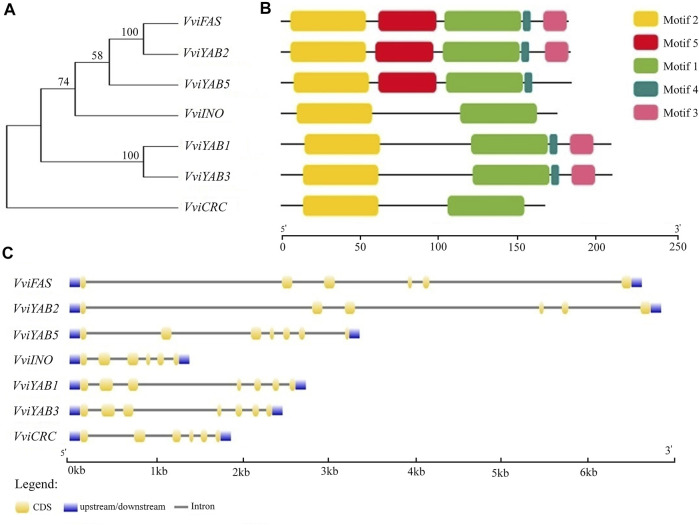
Phylogenetic analysis, gene structure, and conserved motifs of YAB family in grapevine. **(A)** The conserved YABBY domain sequences of VviYAB proteins constructed a neighbor-joining (NJ) phylogenetic tree, and the bootstrap test was performed with 1,000 iterations. **(B)** Distribution of conserved motifs of VviYAB proteins. Different motifs are shown by different colors numbered 1 to 5. See legend for detailed color. **(C)** Exon–intron structure of *VviYAB* genes. Blue indicates untranslated 5′- and 3′- regions, yellow indicates exons; black indicates introns.

All VviYABs contained a C-terminal YABBY domain (Figure 2B and Supplementary Figure S2), which was similar with previously identified YAB proteins. A conserved zinc finger domain was situated near N-terminal (Figure 2B and Supplementary Figure S2). Furthermore, similar protein structures and motif compositions were observed within the groups (Figure 2B and Supplementary Figure S3). For example, motif 3 was present in the C-terminal of YAB2 and YAB1/YAB3 subgroup (Figure 2B). Phylogenetic analysis also showed a close relationship between *VviFAS* and *VviYAB2* and between *VviYAB1* and *VviYAB3* subgroups (Figure 2A), which was consistent with the similarity in motif composition. Moreover, *VviFAS* and *VviYAB2* as well as *VviYAB1* and *VviYAB3* showed similar exon structure (Figure 2C), which further supported the analysis of phylogenetic relationship and motif compositions.

In addition, some of YABBY domains among grapevines, tomatoes, and *Arabidopsis* showed pairwise relationships. A total of 17 pairs of syntenic relations were identified among the three species, including seven grapevine *VviYABs*, five *Arabidopsis AtYABs*, and eight tomato *SlYABs* (Supplementary Figure S4 and Table S2). *VviYAB3*, *AtYAB1*, and *SlYAB1*, belonging to YAB1/YAB5 group, are linked to at least three syntenic events and might indicate a high conservation of group YAB1/YAB3 members. The number of synteny events between grapevines and tomatoes (six synteny events) was greater than that between grapevines and *Arabidopsis* (three synteny events), indicating a high conservation of *YABs* between grapevines and tomatoes.

### Functional Analysis of *Cis*-Elements in the Promoter of *VviYAB* Genes

**FIGURE 3:**
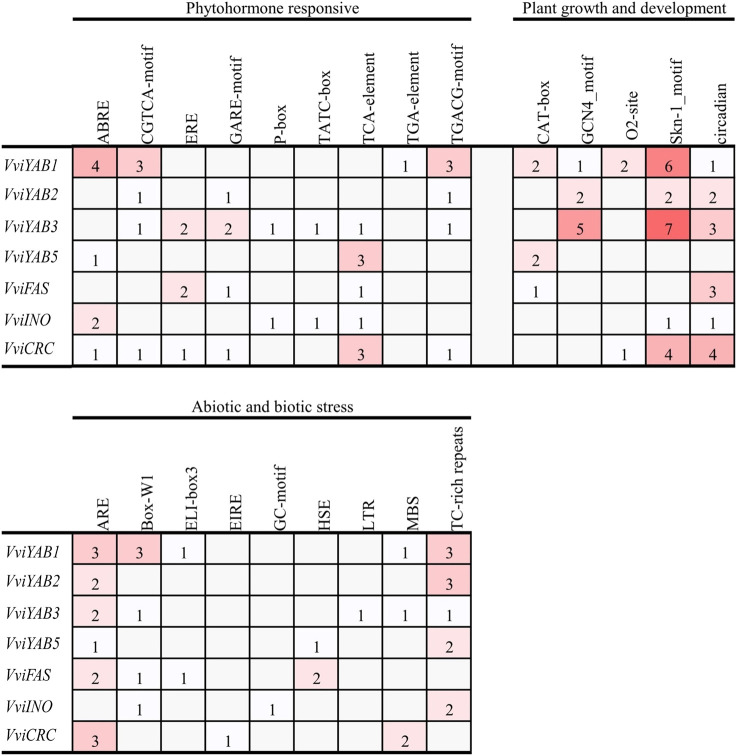
The *cis*-regulatory elements analysis of promoter region of *VviYABs*. The number of each *cis*-acting element in the promoter region (1.5 kb upstream of the translation start site) of *VviYAB* genes. Based on the functional annotation, the *cis*-acting elements were classified into three major classes: plant growth and development, phytohormone responsive, and abiotic and biotic stresses-related *cis*-acting elements.

The evaluation of *cis*-elements in promoters will provide a better understanding of the gene function and transcriptional regulation. As shown in Supplementary Table S3, two basic *cis*-elements, TATA-box and CAAT-box, were widely distributed in the promoters of all *VviYAB* genes. Several growth-related *cis*-elements including the meristem expression element (CAT-box), the endosperm-specific expression element (GCN4_motif and Skn-1_motif), and the circadian regulator element (circadian) were identified in the promoters of three, five, and six *VviYABs*, respectively (Figure 3). In hormone-related *cis*-regulatory elements, the MeJA-responsive element (CGTCA-motif and TGACG-motif), the SA-responsive element (TCA-element), the ABA-responsive element (ABRE), ethylene responsive element (ERE), GA-responsive element (GARE-motif, P-box, and TATC-box), and auxin-responsive element (TGA-element) were identified in the promoter region of *VviYABs* (Figure 3 and Supplementary Table S3). Some stress-related *cis*-regulatory elements, which were associated with anaerobic induction (ARE), fungal elicitor (Box-W1), drought (MBS), and stress responsiveness (TC-rich repeats), were found in six, four, three, and five *VviYABs*, respectively (Figure 3 and Supplementary Table S3).

### Expression Patterns of *VviYAB* Genes in Different Tissues

**FIGURE 4:**
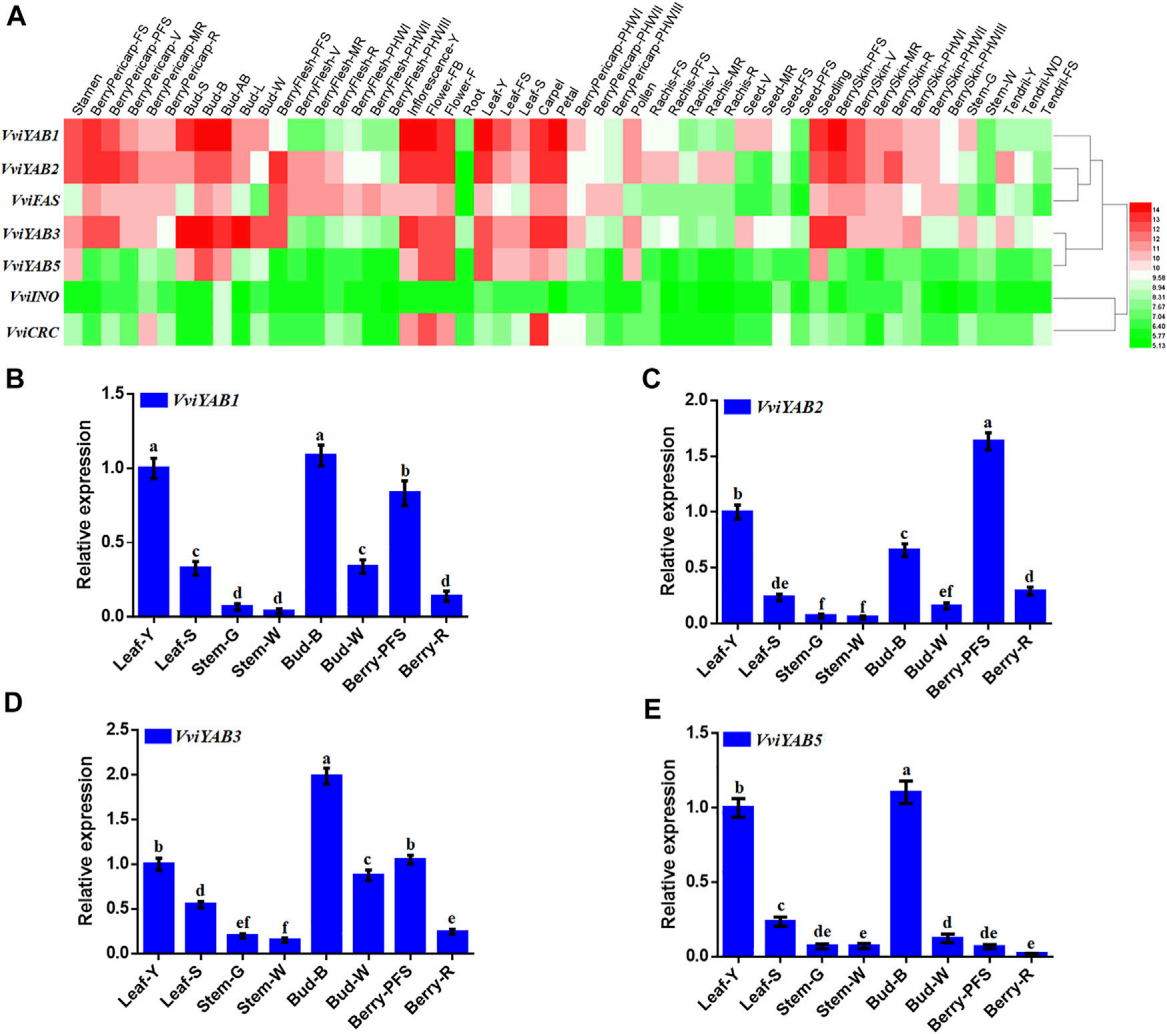
Expression profile of *VviYABs* in various tissues and developmental stages. **(A)** Expression of *VviYABs* in the *V. vinifera* cv “Corvina” atlas. Expression data were normalized based on the mean expression value of each gene in all analyzed tissues. Genes were hierarchically clustered based on average Pearson's distance metric and “average linkage” method. Red and green boxes indicate high and low expression levels, respectively, for each gene. **(B–E)** qPCR validation of *VviYAB* gene expression in four vegetative/green and four mature/woody organs, respectively. Transcripts were normalized to the expression of the *actin* gene. The data are shown as means ± standard deviation (SD) of three replicates. Bud-AB, bud after burst; Bud-B, bud burst; Bud-W, winter bud; Bud-L, latent bud; Bud-S, bud swell; Flower-F, flowering; Flower-FB, flowering begins; FS, fruit set; Inflorescence-Y, young inflorescence with single flowers separated; Inflorescence-WD, well-developed inflorescence; Leaf-FS, mature leaf; Leaf-S, senescing leaf; Leaf-Y, young leaf; MR, mid-ripening; R, ripening; PFS, post fruit set; Stem-G, green stem; Stem-W, woody stem; V, veraison..

To investigate the functions of the *VviYABs*, we estimated their expression levels in 42 various organs/tissues, which were obtained from GEO datasets (GSE36128) by previous microarray analysis (Fasoli et al., 2012). Figure 4A showed that the expression of *VviCRC* was restricted to floral organs, such as well-developed inflorescences and carpels (Figure 4A and Supplementary Table S4), which was in good agreement with previous reports in *Arabidopsis* (Bowman and Smyth, 1999; Villanueva et al., 1999). *VviYAB1* and *VviYAB3* from the same subgroup exhibited high expression levels in leaf-derived organs, including buds, and leaves (Figure 4A and Supplementary Table S4), implying an involvement of subgroup YAB1/YAB3 members in leaf development. Additionally, *VviFAS* and *VviYAB2* showed much higher expression levels in young berries, buds, and floral organs. By comparison, *VvINO* showed lower expression levels in all tested tissues. Remarkably, several *VviYABs*, including *VviYAB1*, *2*, *3*, and *5*, were highly expressed in vegetative/green tissues, implying a potential regulatory function during the developmental transition.

To further understand the expression distinction of *VviYAB* genes between vegetative/green and mature/woody organs by microarray data, the expression patterns of four *VviYABs* were validated by qRT-PCR in four vegetative/green and four mature/woody organs. As expected, qRT-PCR results were highly consistent with the microarray data and showed significantly different expression between vegetative/green and mature/woody samples (Figures 4B–E). For example, all four *VviYABs* showed significantly higher expression levels in vegetative/green organs (young leaf, bud burst, and berry post-fruit set) than in mature/woody tissues (senescencing leaf, winter bud, and berry ripening), in the stem (Figures 4B–E). These results implied that *VviYABs* might be involved in regulatory switch from the immature to the mature developmental phase. Furthermore, four *VviYABs* (*VviYAB1*, *2*, and *3* and *VviFAS*) had higher expression in young berries and then showed a gradual decrease from veraison to ripening stage (Figure 4 and Supplementary Table S4), indicating that these genes might be involved in early fruit development and morphogenesis in grapevines.

### Expression Patterns of *VviYAB* Genes During Berry Development and Ripening

**FIGURE 5:**
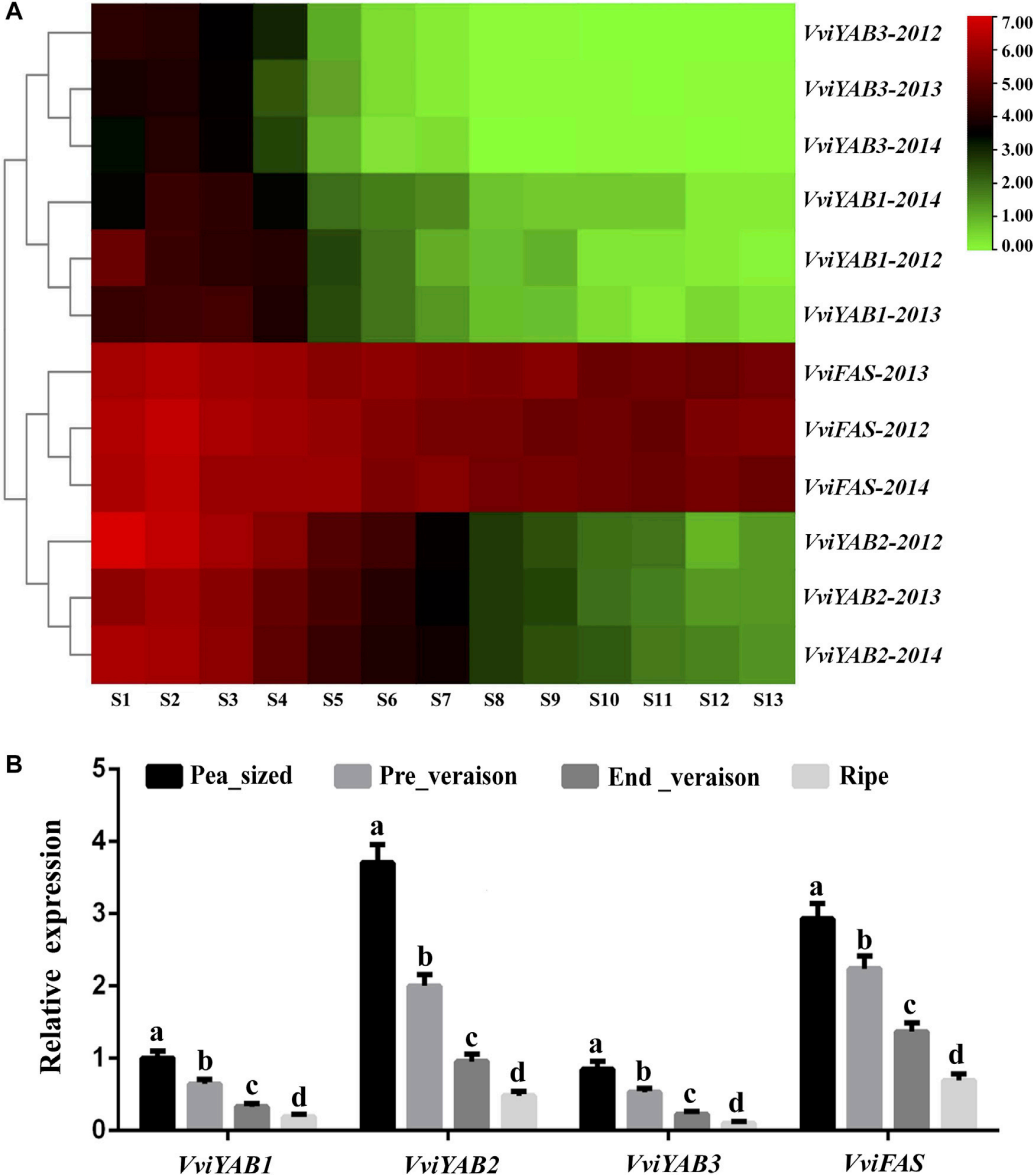
Expression profile of four *VviYAB* genes during developmental and ripening berry in grapevine. **(A)** Expression of *VviYAB* genes in the *V. vinifera* cv “Cabernet Sauvignon” atlas. Berries were collected at 10-day intervals in 2012 and weekly in 2013 and 2014, beginning at fruit set and continuing until harvest (24.5°Brix). S represents stage. Expression data were normalized based on the mean expression value of each gene from fruit set to full maturity. The mean expression values were again normalized using logarithm with the base of 2 using the Heml software. Gene names are displayed to the right of each row. **(B)** qPCR validation of *VviYABs* expression in four berry development stages. Transcripts were normalized to the expression of the *actin* gene. The data are shown as means ± SD of three replicates.

To reveal the putative roles of *VviYABs* during fruit development and ripening, we firstly focused on the expression profiles of *VviYAB* genes from GEO datasets (GSE98923) (Fasoli et al., 2018), which contained 13 different developmental stages from fruit set to full maturity (Figure 5A and Supplementary Table S5). As shown in Figure 5A, the most obvious correlation was that the expressions of four *VviYABs* (*VviYAB1*, *2*, and *3* and *VviFAS*) were negatively related to berry development (from fruit set to full maturity). For example, *VviFAS* and *VviYAB2* showed sharply downregulated expression from immature to the mature berry in grapevines. The expression levels of *VviYAB1* and *VviYAB3* were very similar to those of *VviFAS* and *VviYAB2*, but their expression levels were significantly lower. These results further supported the idea that *VviYABs* promoted the immature-to-mature transition during grapevine berry development. In addition, *VviYAB5*, *VviCRC*, and *VviINO* were always very low or undetected levels during the whole grapevine berry ripening process, indicating that they might not be involved in the regulatory switch during grapevine berry development.

In order to validate regulatory mechanisms involved in the switch between immature and mature berries, qRT-PCR analysis of four *VviYABs* (*VviYAB1*, *2*, and *3* and *VviFAS*) was further carried out at four berry development stages, including pea_sized, pre_veraison, end_veraison, and ripe. We found that qRT-PCR results were completely consistent with those of RNA-seq data (Figure 5B). All four *VviYABs* were highly expressed in immature berries and showed a significant decrease in mature berries, indicating a regulatory switch during berry development and ripening. To obtain more information of *VviYABs* during berry development transition, the transcription accumulation patterns among 10 different grapevine varieties were obtained from previous transcriptome sequencing data (GSE62744 and GSE62745), which also included four different berry developmental stages (Massonnet et al., 2017). The expression of three *VviYABs* (*VviYAB5*, *VviCRC*, and *VviINO*) were not detected during the entire grapevine berry ripening process (Supplementary Figure S5), which corresponded with the data from a previous RNA-Seq analysis (Figure 5A). *VviYAB1*, *2*, and *3* and *VviFAS* remained relatively highly expressed in immature berries (pea-sized berry and pre_veraison stage) and decreased gradually in mature berries (end_veraison and ripe stage) (Supplementary Figure S5). All these results implied that these four *VviYABs* might play fundamental shift roles from the immature to mature berry developmental processes.

### *VviYABs* in Response to Exogenous ABA, Ethylene, GA_3_, and CPPU Hormones

**FIGURE 6:**
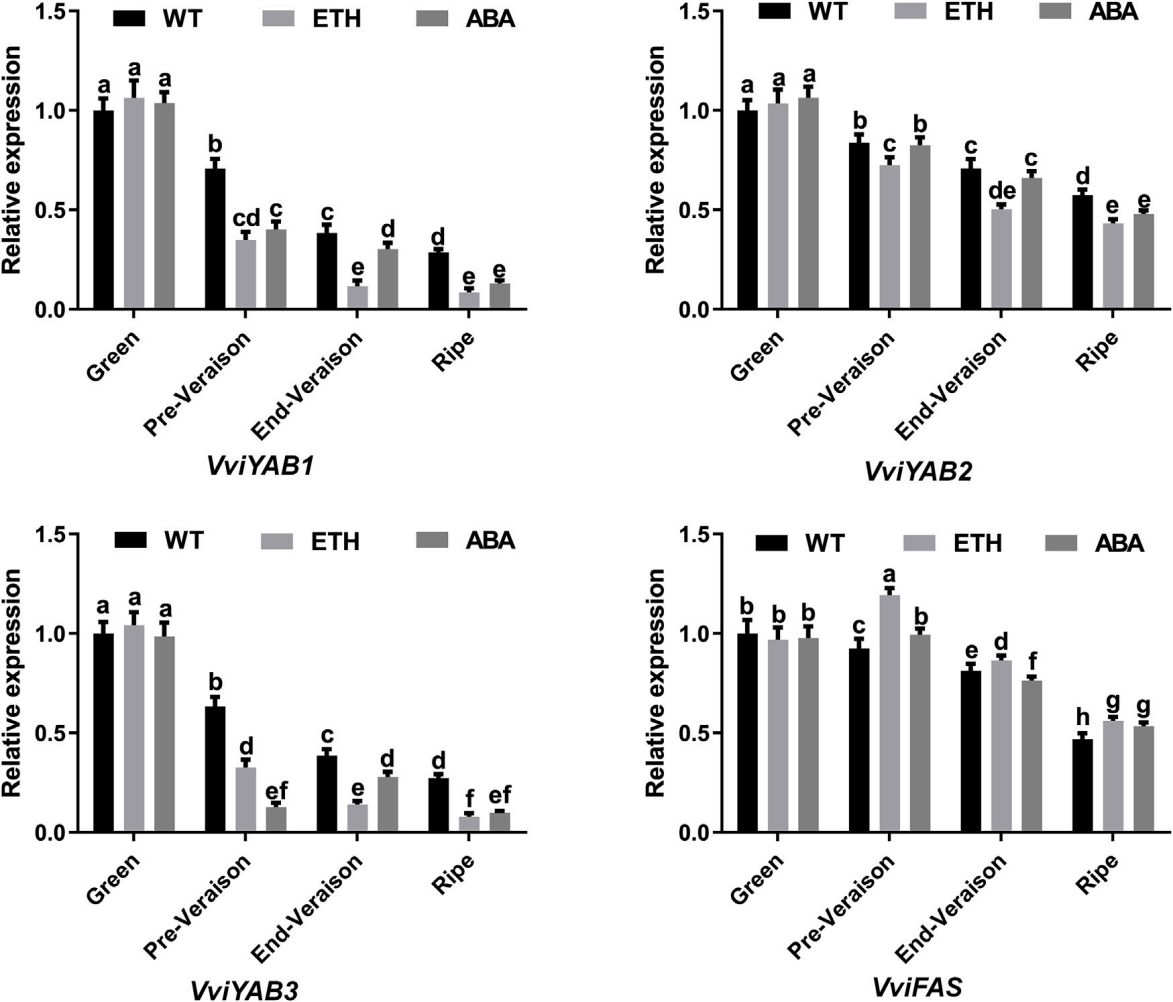
Expression profiles of *VviYAB* genes in response to ABA and ethylene treatments. Transcripts were normalized to the expression of the *actin* gene. The data are shown as means ± SD of three replicates.

To date, the role of YAB proteins in hormonal signaling pathways is scarce in grapevines. To reveal the potential roles of the *VviYABs* in response to ABA and ethylene treatments, four members (*VviYAB1*, *2*, and *3* and *VviFA*S) with high expression levels of *VviYABs* were analyzed by qRT-PCR (Figure 6). Our results demonstrated that ABA and ethylene treatments significantly suppressed the expression levels of *VviYAB1* and *VviYAB3* from Pre-Verasion to ripening period. *VviYAB2* was markedly repressed from pre-verasion to ripening period following the enthylene treatment, but it did not respond markedly to the ABA treatment at pre-veraison and end-veraison periods. However, *VviFAS* was notably activated by enthylene treatment from pre-verasion to ripening period. In addition, the expression of *VviFAS* was significantly induced at pre-verasion and ripening periods, while it was inhibited at the end-veraison stage following the ABA treatment (Figure 6). Interestingly, the four *VviYABs* (*VviYAB1*, 2, and 3 and *VviFAS*) did not markedly differ in the presence of enthylene and ABA at the green berry period.

Currently, GA_3_ and CPPU are usually used conducted to induce seedless fruit set, increase fruit size, and inhibit fruit russet (Xu et al., 2019). Therefore, we used previously published RNA-seq data to investigate in depth the crucial role of the *VviYABs* in response to GA_3_ and CPPU (Xu et al., 2019). As shown in Supplementary Table S6, the expression of *VviYAB1* was nearly unchanged by GA_3_ treatment alone. When GA_3_ in combination with CPPU were applied, *VviYAB1* expression was significantly increased suggesting an involvement of *VviYAB1* in fruit expansion by mediating cytokinin signaling pathway. Furthermore, *VviYAB3* expression was significantly increased by both GA_3_ and GA_3_ + CPPU treatments, implying that it may participate in a more complex regulatory network.

### *VviYABs* in Response to Different Abiotic and Biotic Stresses

**FIGURE 7:**
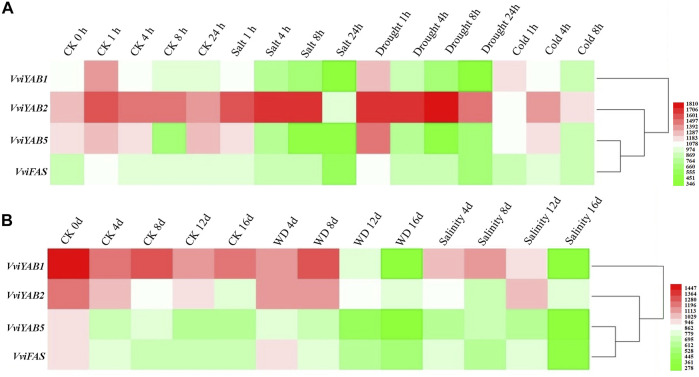
Expression patterns of *VviYAB* genes in response to abiotic stresses. Microarray analysis of *VviYAB* genes in the *V. vinifera* cv. Cabernet Sauvignon were downloaded from the NCBI GEO datasets (GSE31594 and GSE31677) and graphically represented with MeV software. **(A)** *V. vinifera* cv. Cabernet Sauvignon plants grown in a hydroponic drip system were treated with 120 mM salt, polyethylene glycol (PEG), cold (5 °C), or untreated. Shoots with leaves were collected at 0, 1, 4, and 8 h for all treatments and at 24 h for all treatments except cold (GEO series GSE31594). **(B)** Potted *V. vinifera* cv. Cabernet Sauvignon in the greenhouse were exposed to a water-deficit stress (WD) by withholding water or a salt stress by watering plants with a saline solution for 16 days. Non-stressed, normally watered plants served as the control for both treatments. Shoot tips were harvested every 4 days (0, 4, 8, 12, and 16 days) (GEO series GSE31677).

Expression data of *VviYABs* against various abiotic stresses, cold, salt, and polyethylene glycol (PEG), were obtained from the NCBI GEO datasets (GSE31594 and GSE31677) (Figure 7 and Supplementary Table S7). *VviYAB1* and *VviYAB5* showed a sharp downregulation in response to short-term salt and drought treatments. *VviFAS* was downregulated after 24 h under short-term salt and drought stress conditions. Conversely, *VviYAB2* upregulated after 8h under drought stress but was strongly repressed after 24 h under salt stress (Figure 7A and Supplementary Table S7). Interestingly, *VviYAB1*, *VviYAB5*, and *VviFAS* were strongly repressed during the final phase of the long-term stress period (16 days after treatment), whereas *VviYAB2* did not respond to either the long-term water or salt stress (Figure 7B and Supplementary Table S7).

**FIGURE 8:**
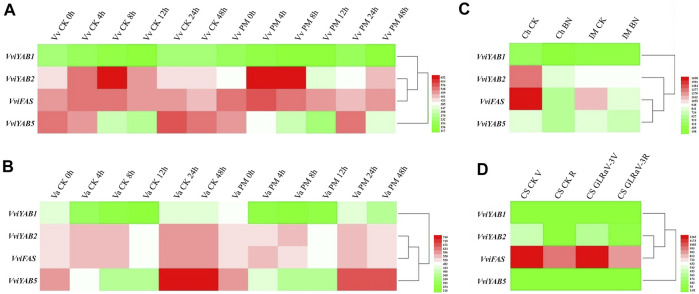
Expression patterns of *VviYAB* genes in response to biotic stresses. **(A, B)** The susceptible variety *V. vinifera* cv. Cabernet Sauvignon” and the resistant variety *V. aestivalis* cv “Norton” plants were grown in an environmental chamber and inoculated with *Erysiphe necator* conidiospores (PM). Inoculated leaves were harvested at 0, 4, 8, 12, 24, and 48 h after inoculation (GSE6404). **(C)** Field-grown plants of *V. vinifera* cv. Chardonnay (Ch) and Incrocio Manzoni (IM) naturally infected with *Bois Noir* phytoplasma (BN), compared to healthy samples (GSE12842). **(D)** *V. vinifera* cv. Cabernet Sauvignon (CS)” was infected with GLRaV-3 during veraison (CS GLRaV-3V) and ripening (CS GLRaV-3R) stages of berry development (GSE31660).

Expression profiles of *VviYABs* were also compared in response to three host-pathogen interaction experiments, such as *Bois Noir* phytoplasma (GSE12842), *Erysiphe necator* (GSE6404), and leaf-roll-associated virus-3 (GLRaV-3) (GSE31660) (Fung et al., 2008; Albertazzi et al., 2009; Vega et al., 2011). The expression aboundance of *VviYAB1* was always lower than *VviYAB2*, 5 and *VviFAS* after inoculation either in cv. Cabernet Sauvignon (susceptible variety) or in cv. Norton (tolerant variety). The transcriptional abundance of *VviYAB5* in response to *E. necator* infection showed a slightly decrease in cv. Norton and Cabernet Sauvignon (Figures 8A, B and Supplementary Table S8). Under *Bois Noir* phytoplasma, four *VviYABs* exhibited significantly decreased expression in the susceptible variety (cv. Chardonnay) than that of tolerant one (cv. Incrocio Manzoni) (Figure 8C and Supplementary Table S9). Finally, four *VviYABs* showed a slightly downregulated expression after GLRaV-3 infection in veraison stage (Figure 8D and Supplementary Table S10).

To obtain more information and validate the expression pattern, other RNA-seq data were collected from four previous studies (Haider et al., 2017; Guan et al., 2018; Zhu et al., 2018). Under drought stress, the expression levels of four *VviYAB* genes (*VviYAB1*, *2*, *3*, and *5*) were significantly downregulated (Supplementary Figure S6 and Table S11), which is similar with the previously published expression files, indicating that *VviYAB1* and *5* were strongly repressed by short- and long-term drought stress periods (Figure 7). In response to waterlogging stress, five *VviYABs* (*VviYAB1*, *2*, *3*, *5*, and *VviFAS*) a decreasing expression trend (Supplementary Figure S6 and Table S11). In addition, *VviYAB1, 2, 3* and VviFAS showed different degrees of reduction under salt microarray and RNA-seq data (Supplementary Figure S6 and Table S11). These results suggest that *VviYABs* play important roles in the response different abiotic and biotic stresses.

### Subcellular Localizations

**FIGURE 9:**
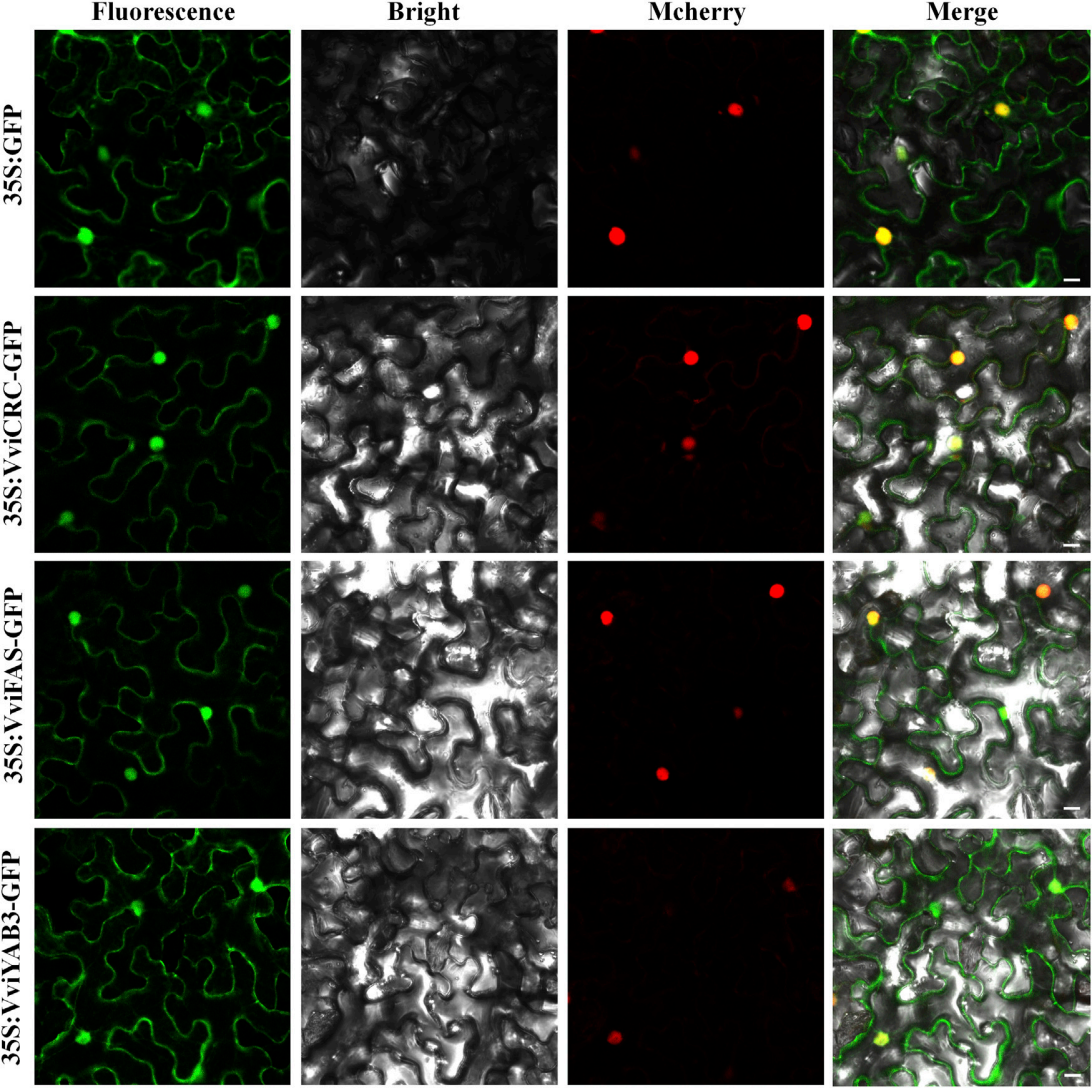
The leaves of 3-to 5-week-old *Nicotiana benthamiana* plants were transiently transformed with control, 35S:VviCRC-GFP, 35S:VviFAS-GFP, and 35S:VviYAB3-GFP. The NLS-mCherry tagged red fluorescent protein was co-expressed. Bar: 25 μm.

The subcellular localization of proteins is desirable for exploring their biological functions. To investigate the *VviYAB* functions, their subcellular localizations were authenticated using the fluorescent protein-tagging method. Firstly, the full-length open reading frames (ORFs) lacking the stop codon of three *VviYABs* were merged to the N-terminal the GFP driven by CaMV 35S promoter, generating fusion proteins 35S-VviCRC-GFP, 35S-VviFAS-GFP, and 35S-VviYAB3-GFP which were agroinfiltrated into leaves of 3- to 5-week-old *N. benthamiana* plants. The vector containing GFP alone (35S: GFP) was used as a control. Fluorescence microscopy exhibited that the control was uniformly dispersed throughout the cell, whereas 35S-VviCRC-GFP, 35S-VviFAS-GFP, and 35S-VviYAB3-GFP fusion proteins were detected in the nucleus and cytoplasm (Figure 9). The results demonstrate that VviCRC, VviFAS, and VviYAB3 are distributed in the cell nucleus and cytoplasm, suggesting their functional similarity.

## Discussion

### Evolution and Structure of the *VviYABs* Family in Grapevines

Plant-specific YAB family plays an important role in abaxial cell development and lateral organ growth (Golz et al., 2004). In addition, YABs have been implicated in plant morphogenesis and stress response. Previous genomic studies have shown that there are 6 YAB members in *Arabidopsis*, 8 in rice, 9 in tomato (Huang et al., 2013), 12 in pakchoi (Hou et al., 2019), and 17 in soybean (Zhao et al., 2017). A total of seven *VviYABs* were identified from the grapevine genome, indicating that the grapevine *YAB* gene family lacked expansion during evolution.

Furthermore, 30 YAB proteins from four plant species, including both dicotyledons and monocotyledons, were divided into five subfamilies according to evolutionary relationships and structural features (Figure 1). As expected, *VviYAB* proteins showed a closer relationship to dicots than to monocots (Figure 1), suggesting that the YAB family was functionally diversified during the evolution of monocots and dicots. Genes from the same subfamily, such as *VviYAB1* and *VviYAB3*, or *VviFAS* and *VviYAB2*, showed similar motif compositions and exon–intron structures (Figure 2), indicating that these gene pairs may have similar functions.

Gene duplication is the primary force that determines the evolutionary mechanisms (Cannon et al., 2004). No segmental and tandem duplication was identified in grapevine YAB family. A total of 17 pairs of syntenic relations were identified among grapevine, tomato, and *Arabidopsis*. Furthermore, the orthologous genes commonly share a similar structure and biological function. Tomato *FAS* is strongly associated with fruit shape classification. Grapevine *VviFAS* was found to share a collinearity relationship with tomato *FAS* genes, suggesting that they probably possess similar functions.

### Potential Roles of *VviYAB* Genes Involved in the Immature-to-Mature Transition

*YAB* genes regulate plant growth, development, and morphogenesis, such as floral organ development and fruit morphology (Chen et al., 1999; Monforte et al., 2014). The expression pattern of *AtCRC* was mostly restricted to carpels and nectaries, while *CRC* plays a critical role in carpel morphogenesis, floral determinacy, and nectary specification in *Arabidopsis* (Bowman and Smyth, 1999)*.* Moreover, *CRC* orthologs were found to be involved in leaf vascular development and carpel identity specification in *Poaceae* (Fourquin et al., 2014). In grapevines, *VviCRC* was preferentially highly expressed in well-developed inflorescences and carpels, which was consistent with the expression profile reported in *Arabidopsis* and *Poaceae* (Bowman, 2000; Fourquin et al., 2014), signifying that *CRC* and its orthologs may perform conserved functions during evolution after speciation. The *AtINO* gene is essential for the growth of the outer integument of the ovule (Villanueva et al., 1999; Simon et al., 2012). Moreover, a previous report showed that *VviYABs*, including *VviINO* gene were expressed at relatively high levels during ovule growth of seedless cultivars, indicating that they participate in the development of ovules (Zhang et al., 2019). However, our data demonstrated that *VviINO* exhibited low expression level in tested tissues of seeded cultivars, which was inconsistent with the findings of Zhang et al. (2019). This may be attributed to the difference in ovule development between seedless and seeded grapevine cultivars.

Previous reports demonstrated that YAB transcription factors regulated vegetative and reproductive development in *Arabidopsis* (Siegfried et al., 1999). In this study, most *VviYAB* genes (*VviYAB1*, *2*, *3*, and *VviFAS*) had relatively high expression levels in floral organs, seedling and early-stage fruits, young leaves, and buds, implying that *VviYABs* have conserved functions and are a broad regulator of grapevines and *Arabidopsis*. *VviYAB5* was precisely highly transcribed during an early stage of burst buds and young leaves, which revealed that *VviYAB5* might be involved in the early-stage bud and leaf development. Notably, *VviYAB1*, *2*, *3*, and *5* were strongly downregulated during the transition from vegetative/green to mature/woody development by microarray data and qRT-PCR assay, indicating that these *VviYABs* inhibited the transition to the mature development phase rather than being activated to implement this developmental program. Taken together, the above-mentioned results revealed that *VviYABs* can be considered as putative positive markers in vegetative/green tissues and negative markers of mature/woody tissues.

### Potential Roles of *VviYAB* Genes in Berry Development and Morphogenesis

The development and maturation of grapevine berry is a complex and dynamic process, which is mainly controlled by a series of transcription factor regulatory networks (Kuhn et al., 2014). Previous studies have shown that *FAS* and *LOCULE NUMBER (LC)* control the number of fruit locules, which ultimately influence both fruit shape and size (Rodríguez et al., 2011; Azzi et al., 2015). *SlYAB2*, the homologous gene of *FAS*, showed differential gene expression between small- and medium-sized tomato fruit varieties (Bartley and Ishida, 2003). In grapevines, the expression of *VviYAB2* was repressed in the *fleshless* berry mutant, which showed significantly reduced fruit size due to a lack of pericarp development (Fernandez et al., 2006; Fernandez et al., 2007). In the present study, *VviYAB2* and *VviFAS* were strongly expressed in early stages of berry development and then decreased dramatically during berry development and ripening (Figure 6), suggesting that these members were important candidates for studying the diversification of berry morphogenesis. Similar with *VviYAB2* and *VvFAS*, but to a lesser extent, the expression patterns of *VviYAB1* and *VviYAB3*, two members of the YAB1/YAB3 subgroup, were also negatively related to berry shape or size. Similarities of these expression patterns indicated that these *VviYABs* were likely to have similar functions with *SlFAS*, which could regulate berry shape or size.

In addition, these four *VviYABs* (*VviYAB1*, *2*, and *3* and *VviFAS*) also showed a significant decrease in mature berries and marked the berry developmental transition from immature to mature. These *VviYABs* can also be considered as putative stage-specificgrapevine berry biomarkers and were used to identify important developmental and metabolic processes in different conditions and organisms.

### Potential Roles of *VviYAB* in Response to Various Phytohormone in Grapevines

Both ethylene and ABA are likely to play important roles, and their interplay may be required to control the berry maturation process (Chervin et al., 2004; Kuhn et al., 2014). Previous studies have shown that both ethylene and ABA could promote berry ripening, and the contents of ethylene and ABA have an increase at the ripening phase (Sun et al., 2010; Wheeler et al., 2019). For example, ethylene biosynthesis related genes are upregulated by ABA treatment (Kuhn et al., 2014). A decrease in berry ABA content was observed following 1-methylcyclopropene (1-MCP) treatment, a specific inhibitor of ethylene receptors (Sun et al., 2010). The role of *VviYAB* genes in the exogenous application of ABA and ethylene is still poorly understood. Previous studies havedepicted that three *BcYAB* genes are upregulated by exogenous ABA treatment in Pak-choi (Hou et al., 2019), implying that *YABs* play a potential role in regulating the ABA signaling pathway. In the current investigation, the expression levels of *VviYAB1* and *VviYAB3* were decreased with respect to berry ripening phase, indicating their role as negative regulators of grape berry ripening. However, the expression levels of *VviYAB1* and *VviYAB3* were sharply increased by exogenous GA_3_ and CPPU treatment. It is well known that GA_3_ and CPPU are usually applied to increase berry size in vineyards. The markedly increased expression of *VviYAB1* and *VviYAB3* under the GA_3_ and CPPU treatments further supported by the possibility that *VviYAB* genes might play an important role in grape berry expansion. These findings demonstrate that *VviYABs* are involved in fruit development by multiple mediating hormone signaling pathways.

### *VviYABs* in Response to Abiotic and Biotic Stresses in Grapevines

Transcriptional regulation is a key indicator of plant responses to series of environmental and biotic stresses. Transcription factors bind to specific cis-elements in the promoter region of the target gene, thereby regulating the function of a particular gene. Previous studies have shown that *YABs* are involved in various abiotic stresses. For example, *GmYAB10* were negatively regulated in drought and salinity responses (Zhao et al., 2017). Most of *GhYABs* in cotton are downregulated under drought and salinity stress, indicating that they may be negative regulators of cotton resistances to drought and salt stresses (Yang et al., 2018). In our study, a series of stress response *cis*-acting elements, such as ARE, MBS, and TC-rich, frequently occurred in the promoter regions of *VviYABs* (Supplementary Table S3). These elements are mainly involved in drought, salt, and waterlogging stresses. All *VviYAB* genes contained at least one of the stress response *cis*-acting elements, indicating their potential functions in response to abiotic stresses. For example, five *VviYABs* were downregulated under waterlogging stress, and all of genes contained ARE elements in their promoters. Furthermore, we found that all *VviYABs* were remarkably downregulated under drought and waterlogging treatments, indicating that these genes might negatively regulate drought and waterlogging responses.

Regarding biotic stresses, *VviYABs* showed low response in resistant varieties compared to susceptible genotypes, which was consistent with the previous results that overall changes in the global transcriptome were usually lower in the resistant genotypes (Fung et al., 2008; Albertazzi et al., 2009). After *E. necator* infection, we observed that *VviYAB5* expression showed a slightly decrease in cv. Norton (resistant genotype) and Cabernet Sauvignon (susceptible genotype) (Figures 8A, B and Supplementary Table S8). The results suggested that *VviYAB5* always responded to *E. necator* infection in both susceptible and resistant grapevine varieties, although the strong induction expression of the transcript did not occur under *E. necator* infection.

All *VviYAB* genes (*VviYAB1*, *2*, and *5* and *VviFAS*) were more strongly decreased in susceptible variety Chardonnay than in the resistant variety Incrocio Manzoni (Figure 8C and Supplementary Table S9), indicating their role in plant defense against *Bois Noir* phytoplasma. Additionally, our *VviYABs* were slightly downregulated after GLRaV-3 infection at the veraison stage (Figure 8D and Supplementary Table S10). The current findings show that *VviYABs* mediate the grapevine defense response against a series of pathogenic infections. Although the microarray and RNA-seq data demonstrated that the *VviYAB* genes responded to different stress conditions, further experiments are needed to validate their putative role and the crosstalk between various environmental stresses.

## Conclusion

In this study, seven *VviYABs* were identified, and their evolution relationship and expression patterns were analyzed systematically in grapevines. Numerous *cis*-acting elements and tissue expression analysis indicated that *VviYABs* were involved in complex regulatory networks controlling growth, development, and responses to abiotic and biotic stresses. Notably, *VviYAB1*, *2*, *3*, and *5* showed significantly higher expression levels in vegetative/green organs than in mature/woody tissues, implying the involvement of regulatory switch mechanisms that might stimulate the transition from the immature to the mature developmental phase. Furthermore, the dramatic downregulation expression of four *VviYABs* (*VviYAB1*, *2*, and *3* and *VviFAS*) marked the berry developmental transition from immature to mature program. These findings demonstrated that these *VviYAB* genes can be considered as putative molecular biomarkers between vegetative/green and mature/woody samples and were used to identify important developmental and metabolic processes in grapevines. In addition, the results suggested an involvement of *VviYAB1* in fruit expansion by mediating cytokinin signaling pathway. Microarray and RNA-seq data showed that *VviYABs* play important roles in response to abiotic and biotic stresses. Our results will pave the way for further studies on the functional conservation and divergence in the *VviYAB* gene family and provide potential candidates for fruit shape and plant abiotic stress tolerance in the grapevine germplasm.

## Data Availability

The original contributions presented in the study are included in the article/Supplementary Material, further inquiries can be directed to the corresponding authors.
