# Supplementary material for: Genome-Wide Identification and Expression Analysis of VviYABs Family Reveal Its Potential Functions in the Developmental Switch and Stresses Response During Grapevine Development
Source: Front Genet. 2022 Feb 3;12:762221. doi: 10.3389/fgene.2021.762221 (PMC8851417; doi:10.3389/fgene.2021.762221)
Supplement: Supplementary file 2 [file DataSheet1.docx]

**Supplementary MATERIAL**


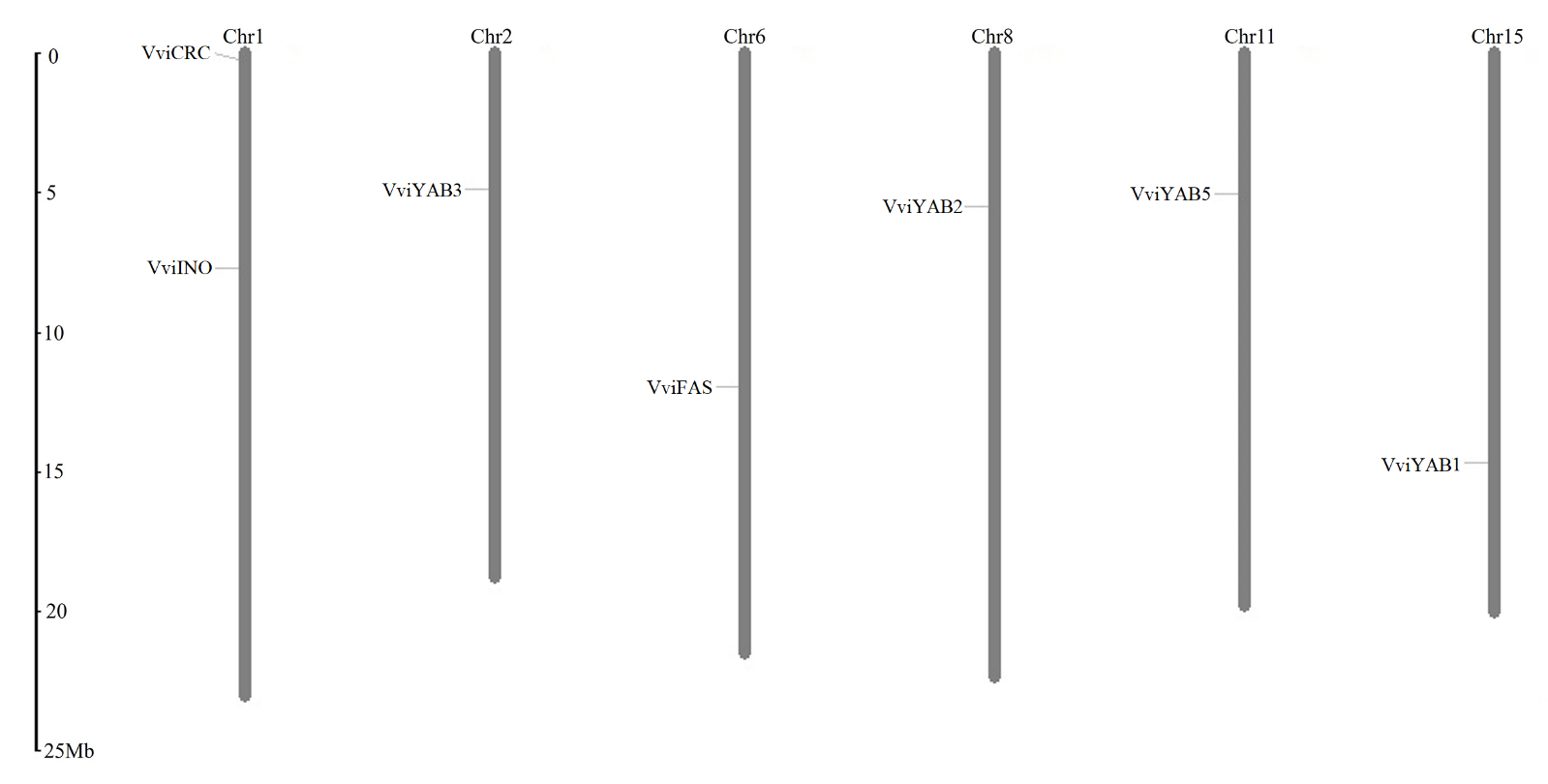


**Figure S1|** Chromosomal distribution of *VviYABs* in grapevine. Chromosome numbers are provided at the top of each chromosome together with the approximate size.


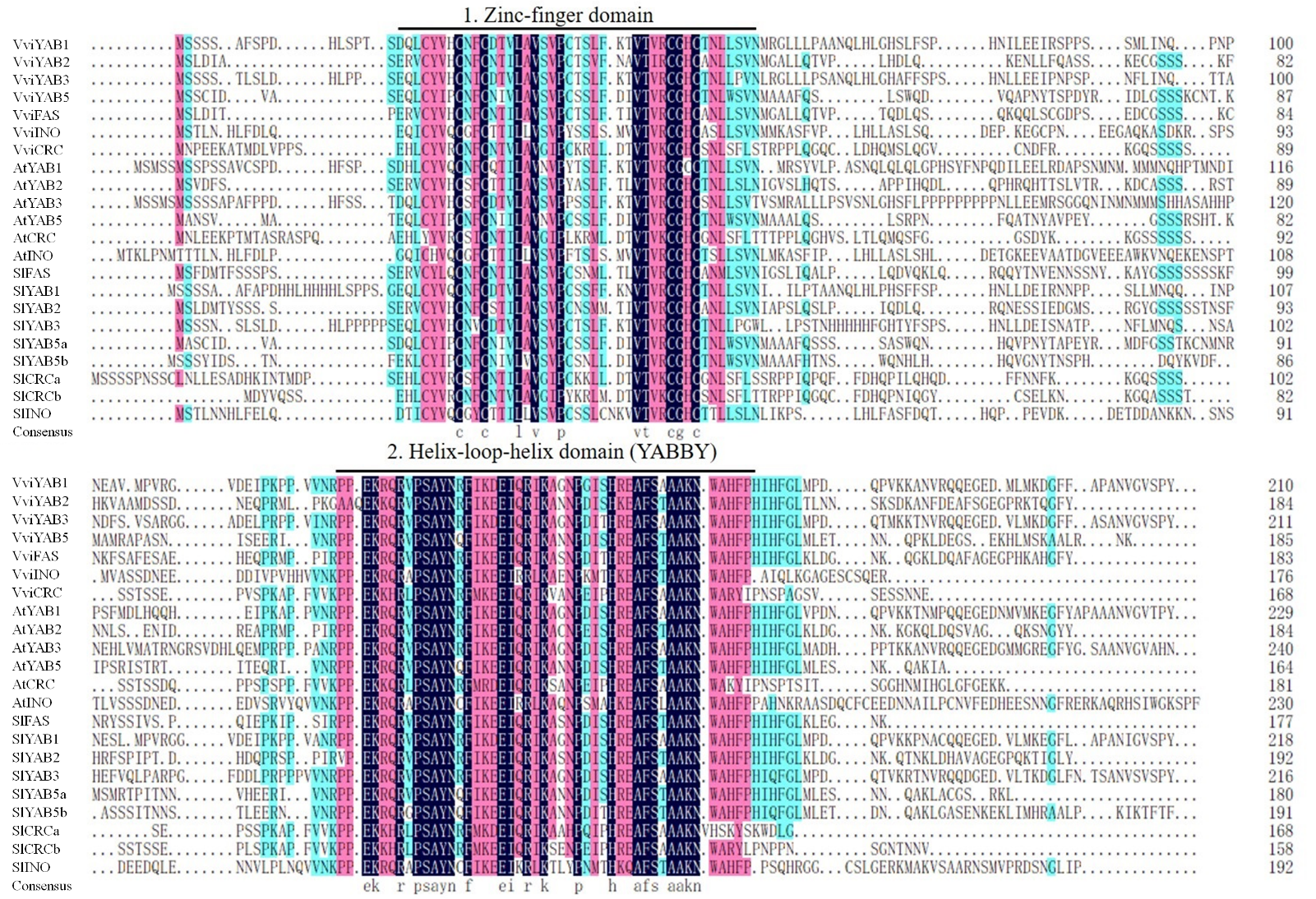


**Figure S2|** Multiple sequence alignment of YAB proteins in grapevine, *Arabidopsis thaliana* and tomato. Sequence alignments were performed using Clustal X 2.0. The zinc finger protein domain and helix-loop-helix (YABBY) domain were indicated above the solid line.


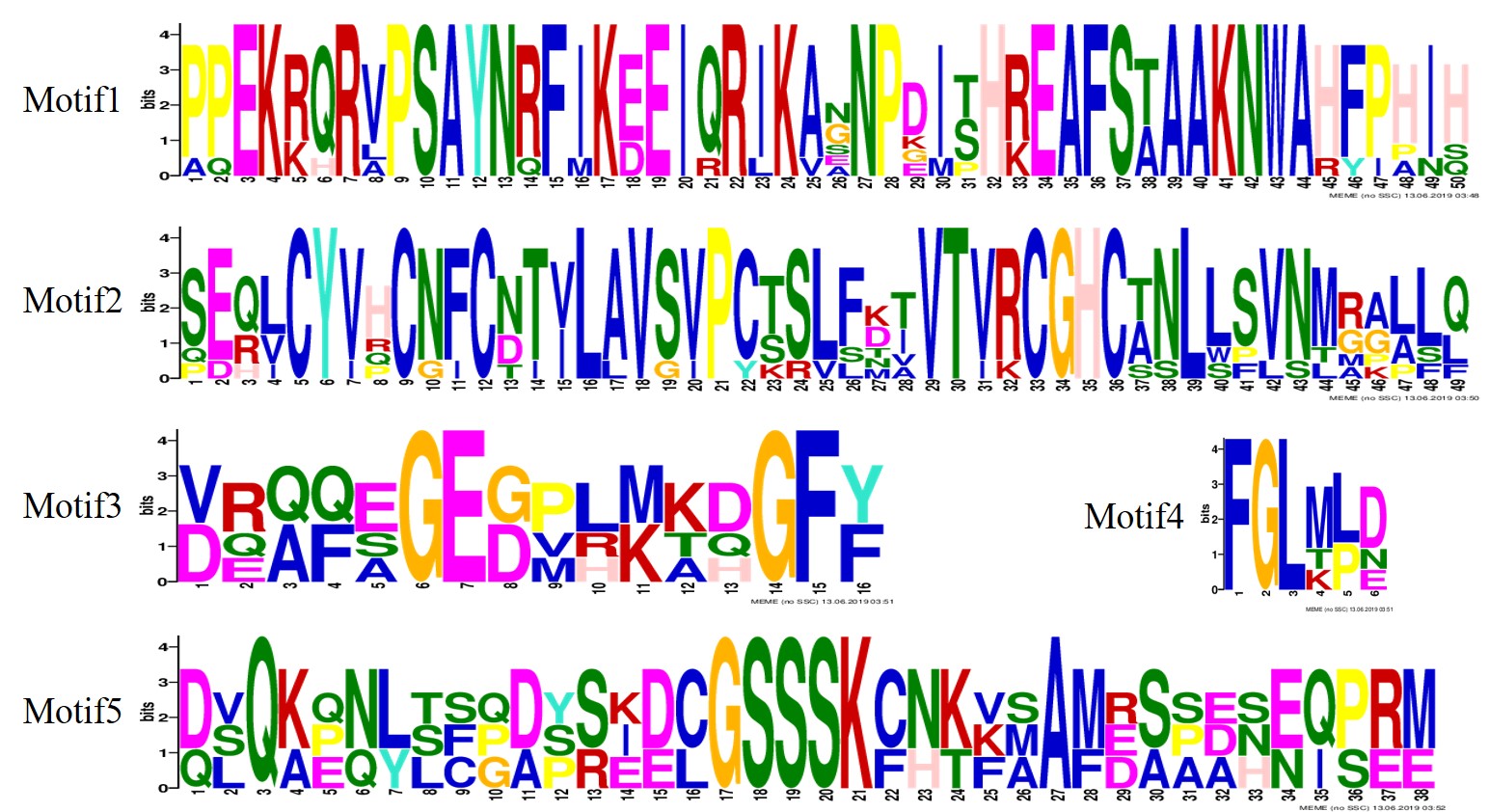


**Figure S3|** The conserved protein motifs in the VviYAB proteins. The *x*-axis indicates the conserved sequences of the domain. The height of each letter indicates the conservation of each residue across all proteins. The *y*-axis is a scale of the relative entropy, which reflects the conservation rate of each amino acid.


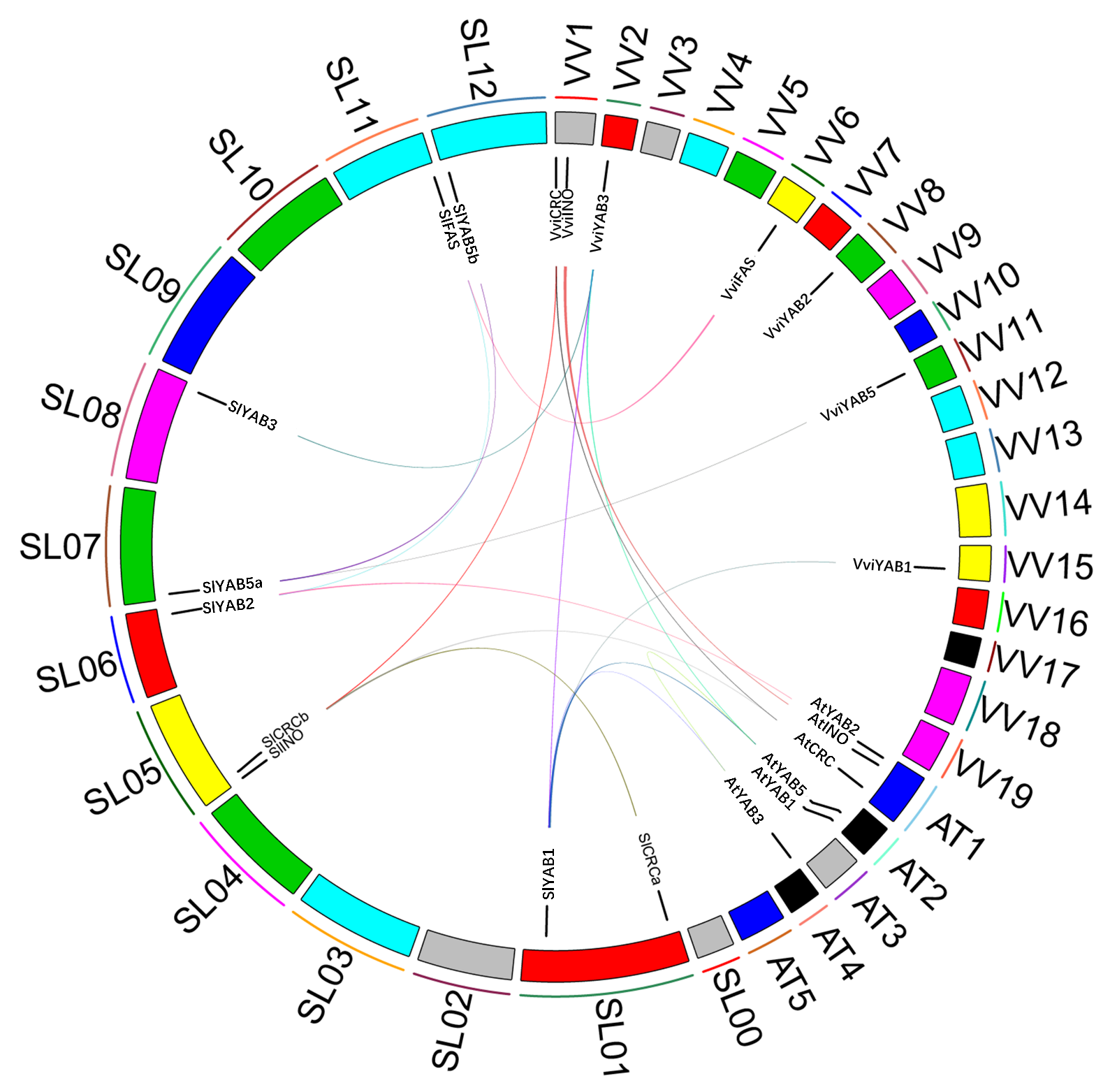


**Figure S4|** Synteny analysis of *YAB* genes among grapevine, *Arabidopsis* and tomato. The chromosomes of grapevine, *Arabidopsis* and tomato are depicted as a circle. The approximate distribution of each *VviYAB*, *AtYAB* and *SlYAB* gene is marked with a short black line on the circle. Colored curves denote the details of syntenic regions among grapevine, *Arabidopsis* and tomato *YABs*.


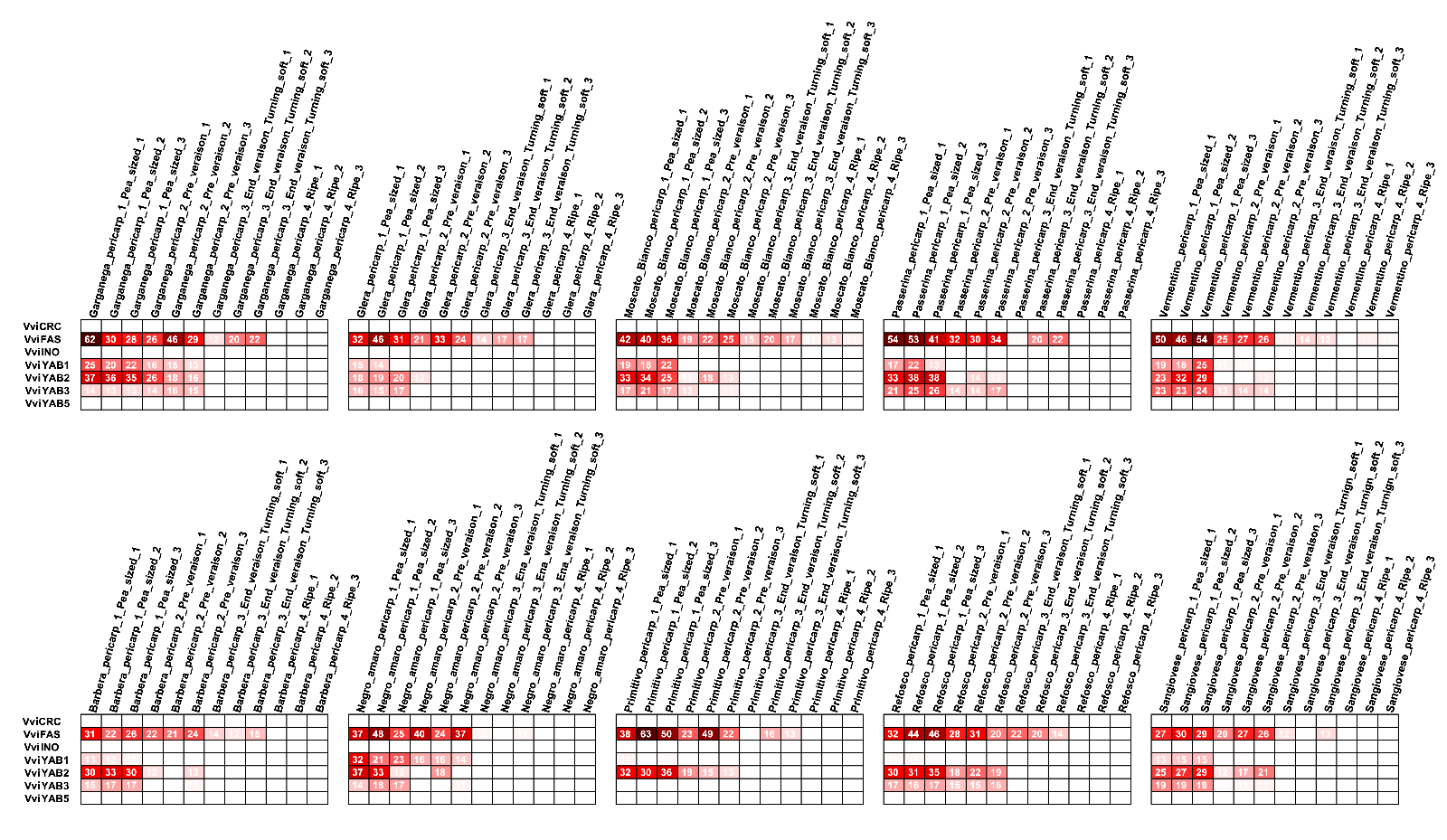
**Figure S5|** Expression profiles of the grapevine *VviYAB* genes in 10 different grapevine varieties at four berry developmental stages. Berries were sampled in triplicate at four developmental stages, the pea-sized berry stage at 20 d after flowering, the berries beginning to touch stage just prior to veraison (Pre_veraison), the berry-softening stage at the end of veraison (End_veraison), and the fully ripe berry stage at harvest.


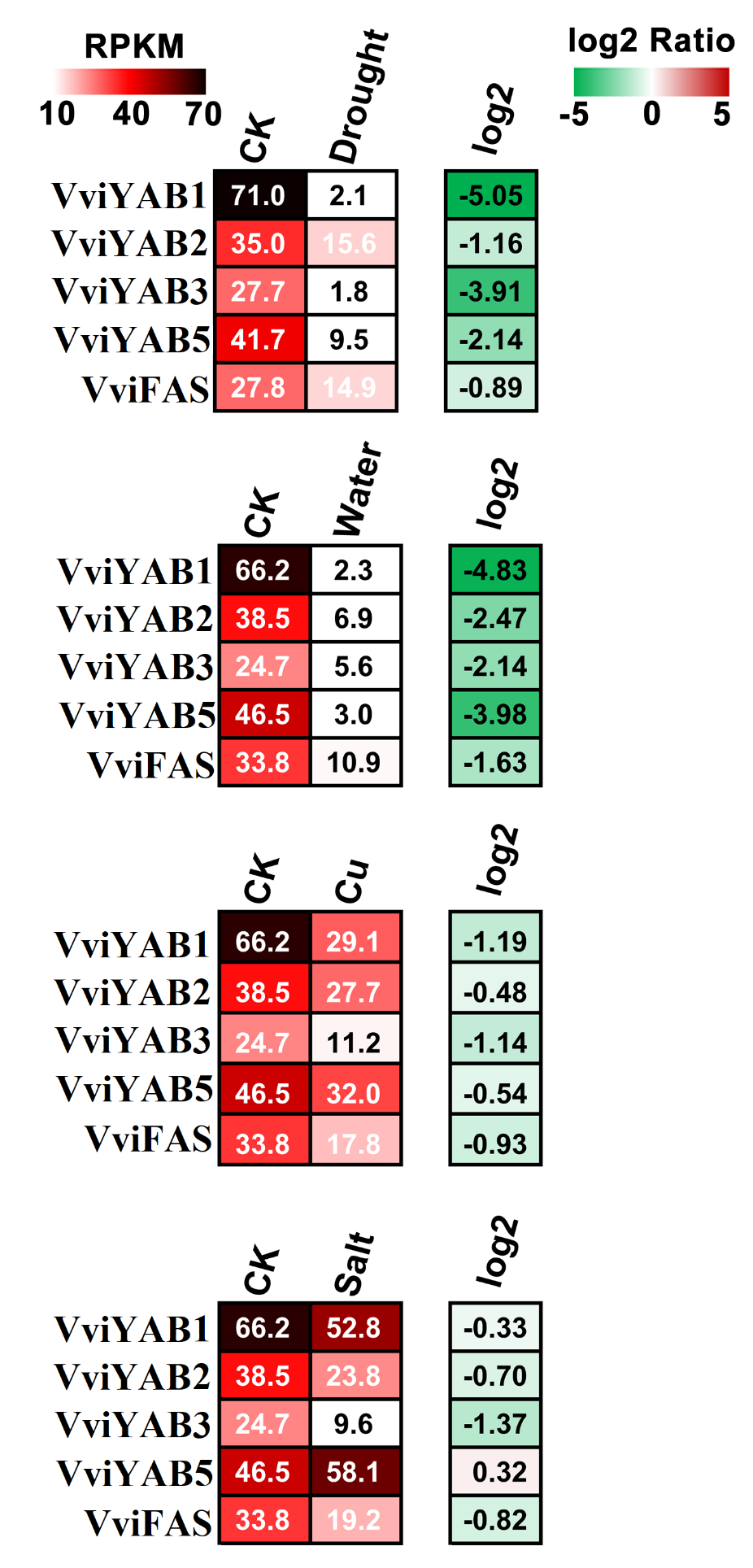


**Figure S6|** The expression pattern of *VviYABs* under drought, waterlogging and salt stresses. Data were obtained by RNA Sequencing and were expressed as Reads Per Kilobase of exon model per Million mapped reads (RPKM). The differentially expressed data were log_2_ transformed with R software. Blocks with green colors indicate decreased and red ones indicate increased transcription levels.
